# Using XAS to monitor radiation damage in real time and post-analysis, and investigation of systematic errors of fluorescence XAS for Cu-bound amyloid-β

**DOI:** 10.1107/S1600576723010890

**Published:** 2024-02-01

**Authors:** Ruwini S. K. Ekanayake, Victor A. Streltsov, Stephen P. Best, Christopher T. Chantler

**Affiliations:** a School of Physics, University of Melbourne, Australia; b The Florey Institute of Neuroscience and Mental Health, University of Melbourne, Australia; c School of Chemistry, University of Melbourne, Australia; Uppsala University, Sweden; The European Extreme Light Infrastucture, Czechia

**Keywords:** X-ray absorption spectro-electrochemical measurements, XAS-EC, radiation damage, dead-time correction, N-truncated amyloid pepides, EXAFS, extended X-ray absorption fine structure

## Abstract

X-ray absorption spectroscopy (XAS) is a promising technique for determining structural information from sensitive biological samples, but high-accuracy X-ray absorption fine structure (XAFS) requires corrections of systematic errors in experimental data. Low-temperature XAS and room-temperature X-ray absorption spectro-electrochemical (XAS-EC) measurements of N-truncated amyloid-β samples were collected and corrected for systematic effects such as dead time, detector efficiencies, monochromator glitches, self-absorption, radiation damage and noise at higher wavenumber (*k*). A new protocol was developed using extended X-ray absorption fine structure (EXAFS) data analysis for monitoring radiation damage in real time and post-analysis. The reliability of the structural determinations and consistency were validated using the XAS measurement experimental uncertainty. The correction of detector pixel efficiencies improved the fitting χ^2^ by 12%. An improvement of about 2.5% of the structural fitting was obtained after dead-time corrections. Normalization allowed the elimination of 90% of the monochromator glitches. The remaining glitches were manually removed. The dispersion of spectra due to self-absorption was corrected. Standard errors of experimental measurements were propagated from pointwise variance of the spectra after systematic corrections. Calculated uncertainties were used in structural refinements for obtaining precise and reliable values of structural parameters including atomic bond lengths and thermal parameters. This has permitted hypothesis testing.

## Abstract 2

X-ray absorption spectroscopy (XAS) measurements of N-truncated amyloid-β samples were corrected for systematic effects such as dead time, detector efficiencies, monochromator glitches, self-absorption, radiation damage and noise at higher wavenumbers *k*, permitting hypothesis testing in structural refinements. A new protocol was developed using extended X-ray absorption fine structure data analysis for monitoring radiation damage in real time and post-analysis.

## 1. Introduction

Many biological systems are investigated using bio-X-ray absorption fine structure (XAFS) because XAFS provides high-resolution interatomic information (Cheng *et al.*, 1999 ▸; Cheung *et al.*, 2000 ▸; Bazin *et al.*, 2014 ▸). However, X-ray absorption spectroscopy (XAS) applications with biological systems are challenging due to the sensitivity of the sample. Achieving high-accuracy XAFS requires standards such as minimization of noise, radiation damage and harmonic contamination, dead-time correction, removal of monochromator glitches, energy calibration, and corrections for self-absorption, detector efficiencies, concentration of sample solutions, and homogeneity of samples and concentration (Chantler *et al.*, 2012*a* ▸; Abe *et al.*, 2018 ▸; Trevorah *et al.*, 2019 ▸). A recent development of the experimental setups enabled accurate XAS measurements of many samples including biological samples and significant improvements in XAFS structural analysis (Chantler *et al.*, 2012*a* ▸; Abe *et al.*, 2018 ▸). However, limitations exist in the determination of the propagation of experimental uncertainties, controlling and evaluating the quality of data and analysis, standardized interpretation of results, and reliability of determined structural information (Chantler *et al.*, 2012*a* ▸; Ascone *et al.*, 2012 ▸; Schalken & Chantler, 2018 ▸; Abe *et al.*, 2018 ▸). Investigating systematic errors, correcting data and hence propagating experimental uncertainty enable precise XAFS analysis (Chantler *et al.*, 2012*a* ▸; Schalken & Chantler, 2018 ▸; Sier *et al.*, 2020 ▸; Ekanayake *et al.*, 2021 ▸).

Standard XAFS analysis returns key parameters for the sample structure, giving insight into the sample coordination geometry, but one disadvantage of standard extended X-ray absorption fine structure (EXAFS) analyses is the inability to provide an absolute determination of a structure or detailed hypothesis testing. This is addressed by employing *eFEFFit* (Smale *et al.*, 2006 ▸; Schalken & Chantler, 2018 ▸) XAFS analysis for the experimental measurements with propagated uncertainties based on systematics because *eFEFFit* returns structural parameters with a reliable quantification. Standard XAFS analysis packages do not incorporate experimental uncertainties (Newville *et al.*, 1999 ▸) in XAFS structural modelling, leading to ambiguity in refinement statistics. Chantler *et al.* (2001 ▸, 2012*b* ▸) introduced techniques to perform transmission and fluorescence XAS experiments to achieve accurate experimental data to propagate systematic uncertainty. Moreover, the Chantler group has implemented experimental uncertainties in XAFS modelling by developing a rigorous XAFS data analysis package (Smale *et al.*, 2006 ▸; Schalken & Chantler, 2018 ▸) to define statistical accuracy in XAFS refinements.

Radiation damage is a key systematic issue in the data collection of electro-generated biological samples. It occurs during the exposure of a sample to an X-ray beam during XAFS data collection. The sample can be oxidized or decomposed due to the absorption of radiation, the damage from heating, the formation of radicals and the breaking of bonds. Potential damage to the samples upon exposure to radiation can be significant and irreversible (Ferraroni *et al.*, 1999 ▸; Kanngießer *et al.*, 2004 ▸; Bertrand *et al.*, 2015 ▸). The most prominent challenge for bio-samples is maintaining the integrity of the system with respect to photodamage (George *et al.*, 2012 ▸). X-ray flux can be reduced by maintaining a cryogenic environment, mainly for dilute biological samples, to control experimental systematics such as photodamage (George *et al.*, 2012 ▸; Sarangi, 2018 ▸). Biological samples are also often available in only small amounts at low concentrations. Frozen samples are used with low X-ray fluxes to control the effect of radiation damage. Electro-synthetic cells and flow cells have been used to obtain *in situ* XAFS of redox states of reactive species (Dewald *et al.*, 1986 ▸; Milsmann *et al.*, 2006 ▸; Wiltshire *et al.*, 2009 ▸; Best *et al.*, 2016 ▸). The collection of a sufficiently large number of quality measurements of samples of *in situ* electrochemically generated states for a considerable duration is a major challenge. The absence of radiation damage was confirmed by making sequential X-ray absorption near-edge structure (XANES) scans and comparing significant XANES feature changes in the study of metal ions in biological samples (Levina *et al.*, 2005 ▸, 2007 ▸). Streltsov *et al.* (2008 ▸) illustrated the deviations in XAS spectra of a Cu-bound amyloid-β_1–16_ (Aβ_1–16_) sample due to radiation damage. Researchers have concluded that radiation damage is inevitable at synchrotrons for fragile proteins at low temperature, so that there is no sense attempting to measure accurate data (Levina *et al.*, 2005 ▸). The density of the photon flux is key to controlling the radiation damage (Bertrand *et al.*, 2015 ▸). The reduction of flux density and faster collection of measurements mitigate the impact of radiation damage. In general, beamlines incorporate filters and attenuators to focus and select the X-ray beam flux relevant to the experiment geometry (George *et al.*, 2012 ▸; Heald, 2015 ▸). The horizontal and vertical adjustment of the beam size controls the flux density on the sample (George *et al.*, 2012 ▸). The concentration of the sample and the experimental geometry, however, limit the potential use of low flux to reduce absorption (Sarangi, 2018 ▸).

High-accuracy XAS is important for investigating structural parameters of Aβ samples. Improvements to the development of our experimental setup enable accurate XAS measurements of Cu-bound Aβ samples at ambient temperatures (Streltsov *et al.*, 2018 ▸; Ekanayake *et al.*, 2023 ▸). The quality of spectra is monitored during the data collection and controlled by adjusting experimental parameters. The elimination and reduction of systematics including monochromator glitches, radiation damage, bubbles in the solution flow, contaminants, inefficiencies in the fluorescence detector, the effect of dead time and normalization of fluorescence spectra are essential for high-accuracy XAFS and for propagating experimental uncertainties.

Investigation of radiation damage is important in a coordination study of metal-bound Aβ. Best *et al.* (2016 ▸) introduced a flow cell to a standard fluorescence XAS experimental setup to obtain XAS measurements of any biological compound such as organometals, proteins and catalysts with minimal radiation damage. They used a low-volume spectro-electrochemical cell, which allows a sample flow through it, to obtain fluorescence XAS under electrosynthesis at ambient temperatures. They collected XAFS data of photosensitive species with minimization of radiation damage and control of redox states. The quality of spectra can be monitored during the data collection and controlled by adjusting experimental parameters.

We have developed a new protocol for monitoring radiation damage using EXAFS data analysis and during the data collection at room temperature. We detail the quality control of Cu-bound Aβ XAS measurements during the experiment and analysis. This paper addresses systematic issues of dead-time correction, deglitching, data truncation, detector inefficiency and normalizing measurements and how to correct for them for sample measurements. The propagation of experimental uncertainties is illustrated. Systematic errors are corrected for, permitting better uncertainties in fitted parameters. This paper gives brief details on the experiment, then considers several major systematics in turn: detector efficiency, dead time, radiation damage (monitoring in real time and post-analysis, and elimination), monochromator glitches in fluorescence, sample heterogeneity in space and time, normalization, and flattening, presented roughly in order of processing.

## 2. Experimental method

XAS was performed on the XAS beamline at the Australian Synchrotron. An X-ray beam was produced with the 1.9 T wiggler with a resolution of Δ*E*/*E* ≃ 1.5 × 10^−4^. A liquid-nitrogen-cooled Si(111) double-crystal monochromator was used to monochromate the beam under Bragg diffraction conditions. A Rh-coated focusing mirror focused the X-ray beam with a harmonic content better than 1 part in 10^5^. The slit size was adjusted to 1 × 1.5 mm to receive an X-ray beam of 1 × 0.25 mm on the sample. The ion chambers were optimized at the beginning of the experiment. The gain of each of the ion chambers was about 10^−9^ and each received about 141 000 counts per second. The dark currents in the upstream and two downstream ion chambers were about 220 965, 1350 and 2230 counts per second, respectively.

Fluorescence XAS measurements were obtained using a 100-element liquid-nitrogen-cooled Ge detector placed 230 mm apart from the samples (Streltsov *et al.*, 2018 ▸; Ekanayake *et al.*, 2023 ▸). Three sample cells, made of polycarbonate, were separated by 6 mm. Approximately 15% glycerol was added to the sample to inhibit the formation of ice. Repeated measurements of each sample were made to increase the measurement statistics (Streltsov *et al.*, 2018 ▸).

**Figure 1:**
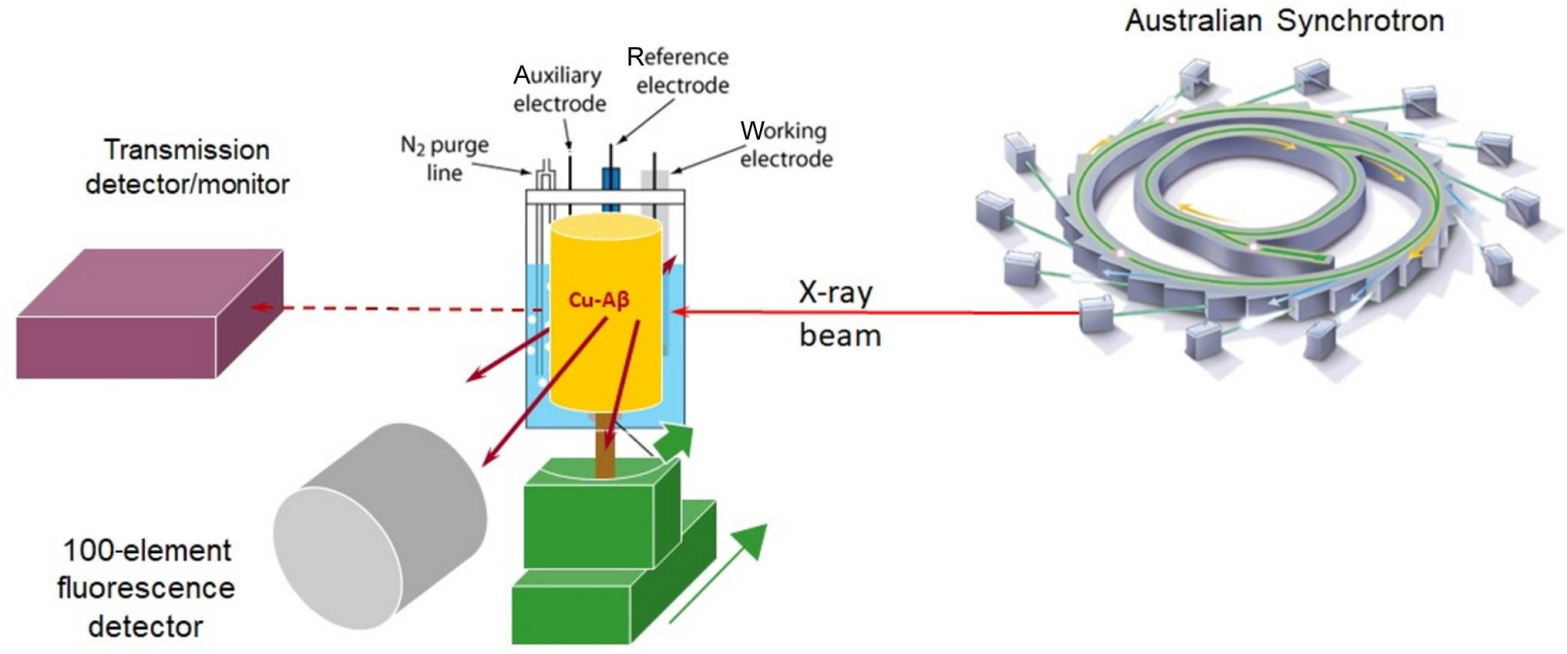
Schematic diagram of the experimental setup used to measure fluorescence X-ray absorption fine structure of copper Aβ peptide with electrochemical control at room temperature under near-physiological conditions (near-neutral pH and under anaerobic conditions at room temperature). The standard electrochemical data do not show any deviance for redox behaviour of Cu ions in amyloid-β. High-accuracy X-ray fluorescence spectra with redox observations were achieved with this novel experimental setup for Cu-bound N-truncated amyloid-β peptides.

Room-temperature XAS experiments were performed under electrochemical control (XAS-EC) using a novel flow electrosynthesis cell (Fig. 1 ▸) (Streltsov *et al.*, 2018 ▸; Ekanayake *et al.*, 2023 ▸). Aβ_4–8_, Aβ_4–12_ and Aβ_4–16_ (F_4_RHDSG_9_­YEV_12_HHQK_16_) sequences of N-truncated amyloid-β peptides were used. Samples can be maintained in the electro-generation stage during the XAS measurements using a cell which permits anaerobic handling of solutions. A small amount of sample is required for collecting XAS spectra as the volume of the cell is small. Because a pulsed flow pattern was applied during electrochemical reactions and throughout the collection of XAS-EC spectra, the contact of the solution with the working electrode (WE) was maximized. Bubbles created in the cells were removed or minimized by a flush controlled by an increase or decrease in potential.

The distance between the samples and the fluorescence detector was set to 120 mm to enable the collection of high-quality room-temperature XAS data. The intensity of the incoming beam was reduced by narrowing the horizontal shutters to 0.6 mm. The intensity counts of the upstream ion chamber were 80 000–90 000 counts per second.

For XAS data collection, aliquots of Aβ peptides were dissolved in a phosphate buffer (PB) of pH 7.4. Then the peptides were complexed with CuCl_2_ (Himes *et al.*, 2008 ▸) at a Cu:peptide molar ratio of 0.9:1. The final concentration of the solution was up to 2 m*M*. Complexes were incubated for about 1 h at room temperature prior to the experiments.

## 3. Detector inefficiency

Each pixel of the detector produces a separate X-ray emission spectrum which is also used to investigate dead pixels and low-quality defective pixels.

**Figure 2:**
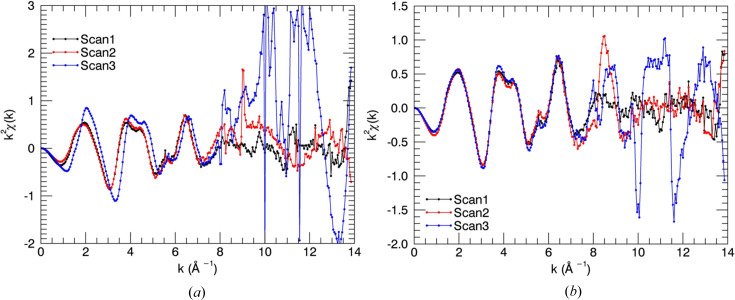
*k* ^2^χ(*k*) spectra of Cu^II^:amyloid-β_4–12_ (*a*) before and (*b*) after excluding dead and defective pixels. Defective pixels destroyed the shape of the oscillations in scan3. The repeated measurements were in good agreement after excluding defective pixels.

**Figure 3:**
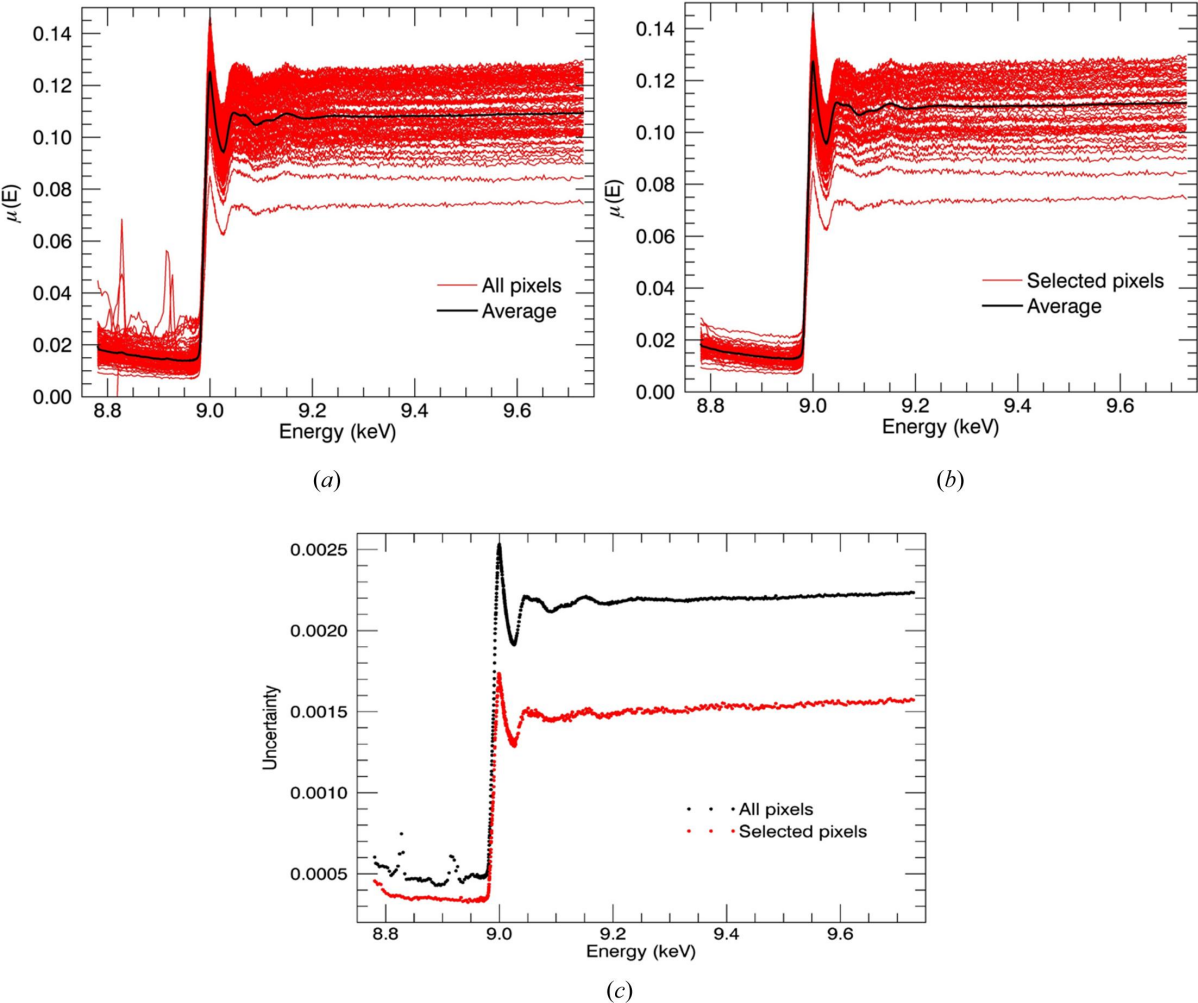
Fluorescence spectra obtained from each pixel in the 100-element detector (red) and their average fluorescence spectrum (black) (*a*) before and (*b*) after exclusion of defective pixels. (*c*) The uncertainties at each energy point before and after excluding defective pixels. The uncertainty of the measurements has been significantly improved by excluding defective pixel data.

The dead pixels which were observed are due to manufacturing faults such as poor bump-bonding and overexposure of pixels. Defective pixel data have low sensitivity or are affected by baseline artefacts. Defective pixels cause spectra with large fluctuations, noise and artefacts. Exclusion of defective pixels and dead pixels from the average was carried out for amyloid-β measurements, after careful examination of individual pixel spectra. Fig. 2 ▸ presents the plots of *k* ^2^χ(*k*) versus wavenumber *k* before and after excluding defective pixels for Cu^II^:amyloid-β_4–12_. The improvement of the spectra is noticeable. Defective pixels destroyed the shape of the oscillations in some scans. High-quality measurements can have a major impact on the structural refinements. An *R* factor of 4.1% and χ^2^ of 98 from the preliminary XAFS structural analysis performed by the *ARTEMIS* software package (Ravel & Newville, 2005 ▸) for the Cu^II^ binding site of amyloid-β_4–16_ and amyloid-β_4–12_ measurements including all pixels was followed by *R* = 3.9% and χ^2^ of 86 excluding dead and defective pixel data (Fig. 3 ▸). We note that many other researchers encounter similar problems with pixel non-uniformity, and also address the issue in a similar manner.

## 4. Dead time and correction

Dead time is an important issue with multi-element fluorescence detectors (Cramer *et al.*, 1988 ▸). It significantly affects the pulse processing electronics. If two events are separated by a very short time, a fluorescence detector cannot process the two events when they reach the detector (Knoll, 2010 ▸; Diaz-Moreno, 2012 ▸). These types of detector systems are referred to as paralysable systems. The uncorrupted throughput rate *R* of radiation for a paralysable system is given by

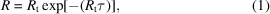

where *R*_t_ is the true input rate to the system and τ is the dead time associated with the system (Knoll, 2010 ▸; Farrow *et al.*, 1995 ▸). Then τ = 2(*t*_p_ + *t*_g_), where *t*_p_ is the energy peaking time, the energy filter length or the integration time and *t*_g_ is the gap time.

Features of the X-ray absorption near-edge region of the absorption spectrum are damped due to the effect of dead time (Zhang *et al.*, 1993 ▸). When the amplitude is reduced, the accuracy of the structural refinements is diminished. The effect of dead time can be reduced by employing sophisticated adaptive signal processing hardware (Farrow *et al.*, 1995 ▸), adding more channels to the detector to minimize saturation (Zhang *et al.*, 1998 ▸) or using pile-up rejection together with the region of interest within the multi-channel analyser (Creagh & Hubbell, 1990 ▸); however, we still need to correct for resulting dead time in most detectors of this type.

The correction for dead time is ignored in some XAFS data analyses, resulting in about 70% amplitude reduction in the XAFS spectrum (Zhang *et al.*, 1993 ▸). However, there are also many experiments that correct for the effect of dead time in XAS (Creagh & Hubbell, 1990 ▸; Creagh, 1987 ▸; Chantler *et al.*, 2012*b* ▸; Sobott *et al.*, 2013 ▸; Woicik *et al.*, 2010 ▸). The dead time should be determined experimentally, especially for systems with multi-channel detectors due to the contribution of pulse-height analysis and storage processes in dead time (Creagh, 1987 ▸). The dead-time correction has been incorporated into all further analysis of copper-binding structures.

**Figure 4:**
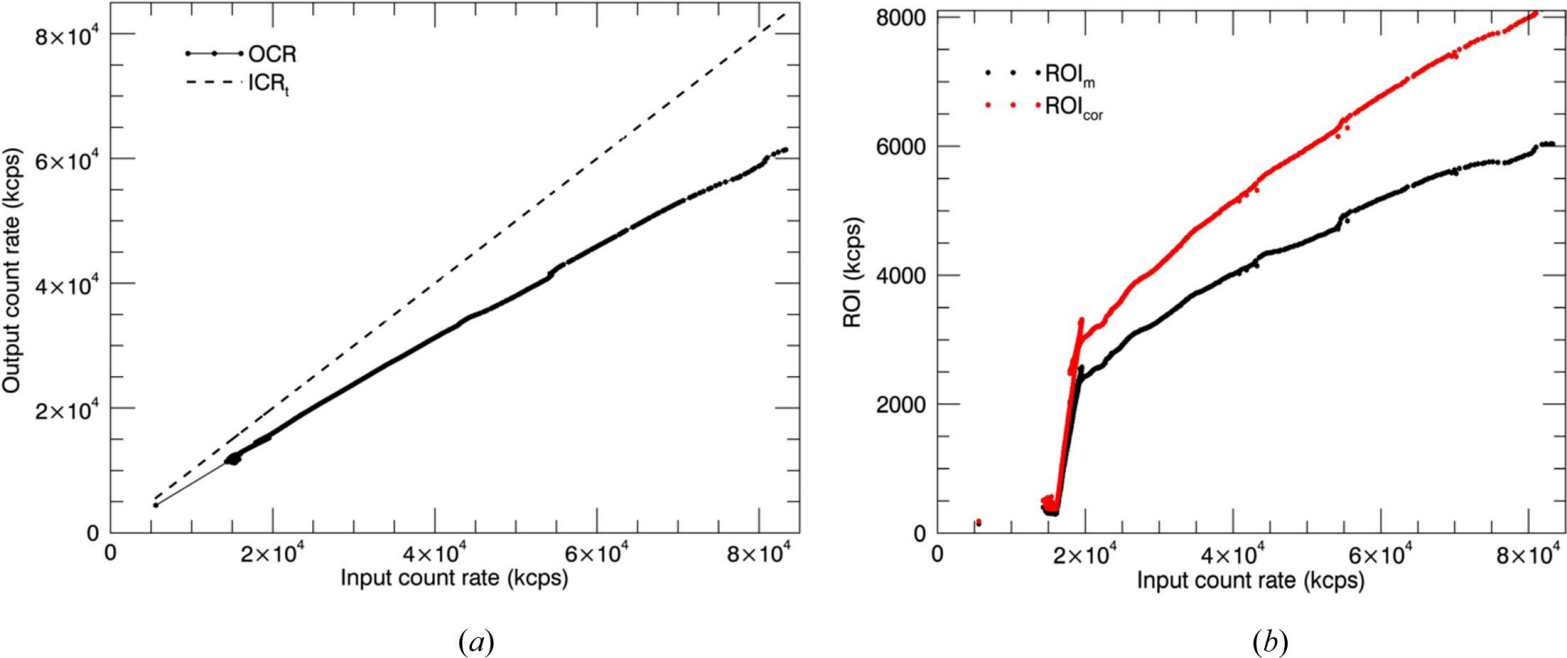
(*a*) OCR versus ICR for detector measurements. Note the nonlinearity of the observed count rate before the correction. (*b*) Count rate in the region of interest versus input count rate obtained for a Cu^II^:amyloid-β_4–12_ sample. Unusual behaviour in the oscillatory pattern disappears after correcting for dead time.

Multi-parameter analysis at high input count rates (ICR) can be carried out from the fast and slow analysis of a digital X-ray processor. In the current work, fast processing signals (ICRs) were obtained prior to measuring energy and similar slow processing signals of all photons tagged (observed current rate, OCR) were obtained after the energy conversion in the energy region of interest (ROI). The OCR output will be generated by 

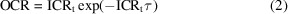

using the dead time [equation (1)]. ICR_t_ is the true incoming count rate. The measured input current rate (ICR_m_) will always be less than the true count rate ICR_t_ because of fast channel pile-up (Woicik *et al.*, 2010 ▸; Abbene & Gerardi, 2015 ▸). Fig. 4 ▸(*a*) presents the values of OCR versus ICR. Note that the OCR deviates from linearity by 25% with increasing ICR. If the count rate is higher, the linearity of the relationship between the counts processed by the detector and the counts reaching the detector will fail and the throughput rate can approach zero (paralysable) (Diaz-Moreno, 2012 ▸). This leads to poor resolution and low-accuracy XAFS.

All the channels are affected by the same dead time for a detector with independent and stochastic pulses (Unonius & Suorttri, 1989 ▸). In the current work, measurements for each detector channel were obtained so that corrections of the results for each detector channel were obtained by implementing the dead-time correction. The dead-time-corrected count rate in the ROI is

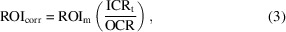

where ROI_m_ is the measured count rate in the ROI. Fig. 4 ▸(*b*) illustrates the uncorrected ROI and corrected ROI against ICR. Unusual behaviour in the oscillatory pattern disappeared after correcting for dead time.

The dead-time-corrected fluorescence spectra are generated by

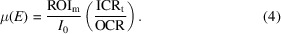

In this XAFS analysis, the dead-time correction for the fluorescence measurements has been implemented using equation (4). The dead-time correction smoothed the data and improved the quality of the spectrum [Fig. 4 ▸(*b*)].

**Figure 5:**
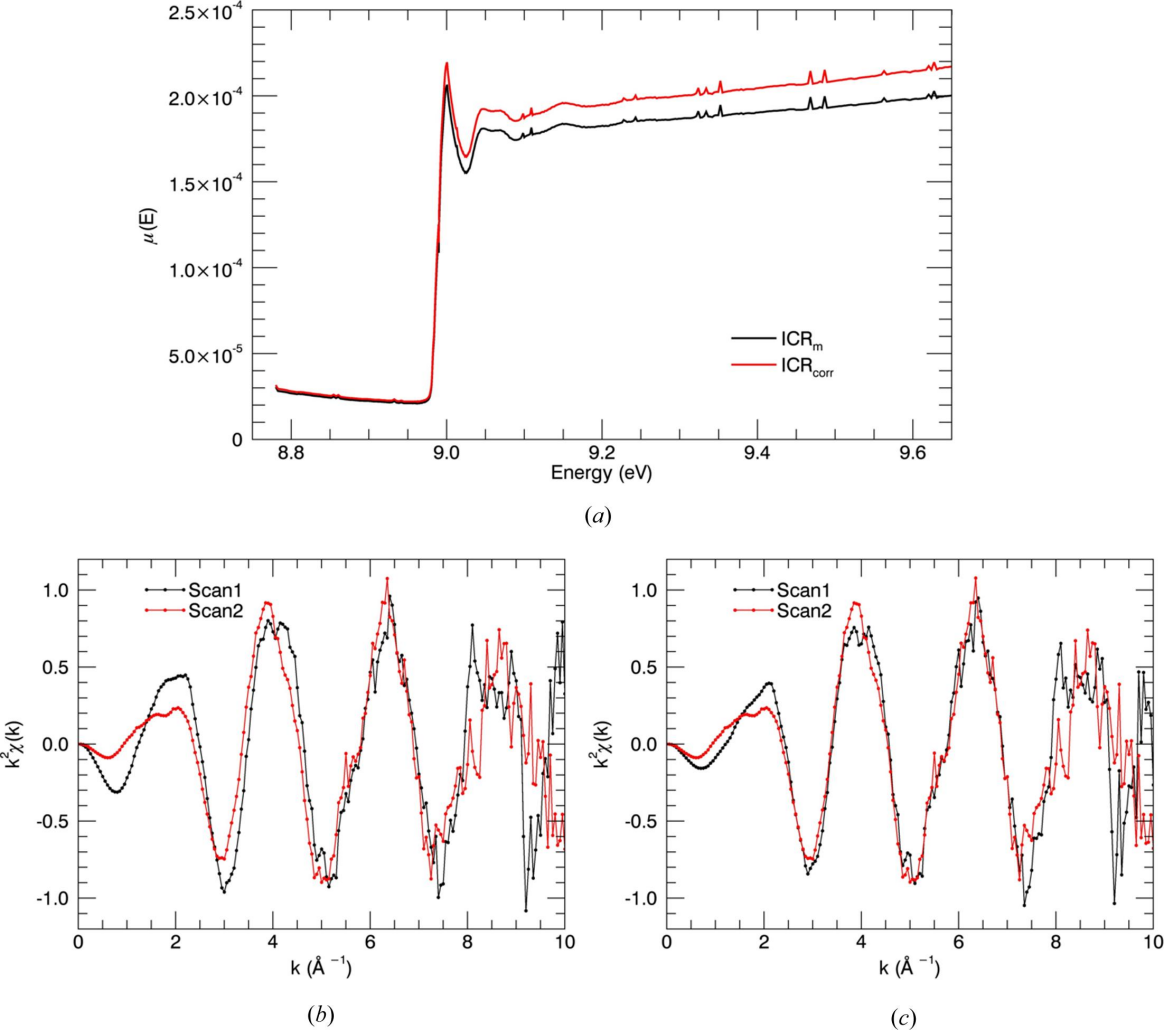
Fluorescence spectrum with dead-time-corrected measurements for Cu^II^:amyloid-β_4–12_. (*a*) Dead-time correction with measured input count rate ICR_m_ (black) and corrected input count rate ICR_corr_ (red) is compared and an 8% change in the measurements after the absorption edge is observed. χ(*k*) plots of scan1 and scan2 with (*b*) ICR and (*c*) ICR_corr_ in dead-time corrections. Repeated scans are consistent when using ICR_corr_.

Correction of the measurements obtained for each detector channel was conducted by implementing the dead-time correction and taking the average of 100 pixel scans. This resulted in an improvement in the signal-to-noise ratio. However, the ICR itself experiences dead-time loss. The importance of using ICR_t_ in determination of corrected count rates in the ROI is discussed by Warburton (2004 ▸), Pushie *et al.* (2014 ▸) and Walko *et al.* (2011 ▸). Ignorance of the true value systematically overestimates the correction. ICR_t_ can be expressed as

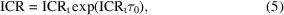

where τ_0_ is the dead time for the ICR channel. The *Sakura* (Kappen *et al.*, 2015 ▸) pre-processing tool used at the Australian Synchrotron includes two options for dead-time correction, outputting ICR or ICR_corr_ in equation (3). Fig. 5 ▸(*a*) illustrates the change in fluorescence spectrum when using corrected ICR measurements in equation (4). There is about an 8% change in the fluorescence spectrum after the absorption edge for uncorrected data. This can affect the accuracy of the structural refinements. Fig. 5 ▸(*b*) and Fig. 5 ▸(*c*) present the repeated χ(*k*) spectra of the sample with ICR and ICR_corr_ measurements. Repeated scans are in good agreement with the use of ICR_corr_ in dead-time corrections. In this context, the combined dead-time processing is relatively unique, and should also lead to further investigation.

An *R* factor of 4.0% with χ^2^ of 86 was achieved from the preliminary EXAFS analysis performed with the *ARTEMIS* software package (Ravel & Newville, 2005 ▸) for the Cu^II^ binding site of amyloid-β_4–16/12_ measurements with the dead-time corrections incorporating ICR, which improved to 3.9% and 86, respectively, for the measurements with the dead-time corrections including ICR_corr_. Use of ICR_corr_ in the XAFS analysis slightly increased the quality of the refinement. Therefore, all the *ARTEMIS* refinements used the dead-time-corrected data with ICR_corr_ measurements. When τ_0_ is unknown, it is assumed that the ICR channel is not affected by the dead-time loss. Then ICR_t_ becomes ICR, treating τ_0_ as 0 s.

## 5. Minimizing and diagnosing radiation damage

Radiation damage (photodamage or photoreduction) depends on the chemical and physical conditions of the sample (Holtona, 2007 ▸) and the amount of incident flux on the sample (George *et al.*, 2012 ▸; Ascone *et al.*, 2012 ▸). Repeated scans are obtained to monitor the radiation damage and repetitions, where the level of photoreduction is negligible, are averaged to obtain the signal-to-noise ratio for determining precise bond lengths (Rich *et al.*, 1998 ▸). Many XAS experiments are performed in a cryogenic environment (Heald, 2015 ▸) to reduce the X-ray beam related radiation damage. In this experiment, the photoreduction of samples at the cryostat was tested by obtaining quick XANES measurements from the same sample position with 30 min exposure intervals.

**Figure 6:**
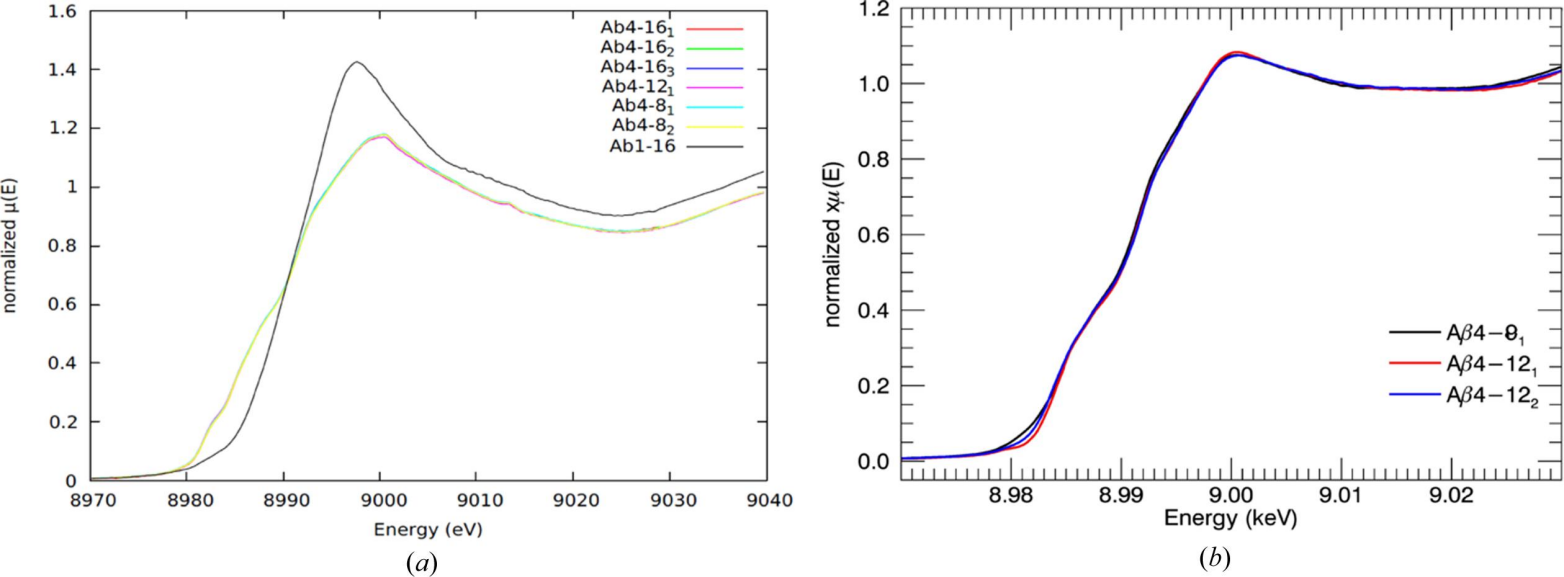
(*a*) XANES spectra of Aβ_4–16_*x*__ (*x* = 1, 2, 3), Aβ_4–12_*x*__ (*x* = 1, 2) and Aβ_4–8_*x*__ (*x* = 1, 2) peptides at low temperature. (*b*) XANES spectra of Aβ_4–8/12/16_ peptides at room temperature. All peptides are consistent with each other at low and room temperature. This result indicates negligible damage to the sample from radiation during the collection of measurements.

Fig. 6 ▸(*a*) shows the repeated XANES spectra of Aβ_4–8/12/16_ peptides. Three repeated measurements at the same position of the sample, *e.g.* Aβ_4–16_*x*__ (*x* = 1, 2, 3), provide similar XANES plots. All three samples illustrate the same XANES features during the exposure. The fact that the plots are consistent provides strong evidence that the low-temperature measurements were associated with negligible beam damage during the experiment. Moreover, XAFS analysis of individual scans was performed using *eFEFFit* to study the existence of radiation damage. Interestingly, the refined measurements including bond lengths and displacement parameters of each individual scan [Table 1 of Ekanayake *et al.* (2023 ▸)] are similar for the same sample, confirming the successful minimization of radiation damage.

The repeated measurements do not show the loss of amplitude and blurring of spectral features expected from radiation damage. If radiation damage permitted other binding configurations (trigonal binding has been reported), we would expect to see developing pleomorphism and blurring of features, but this is not seen in the data.

We performed the two-sample *t*-test to statistically measure the consistency of sample measurements with minimization of radiation damage. Here, low-temperature XAS fluorescence measurements of two different scans for the same sample were considered. We assumed that the measurements are distributed normally and tested the hypothesis that the measurements are similar due to the minimization of radiation damage. The *t*-score is −0.061. If equal variances are assumed, then the number of degrees of freedom is 1274. The critical value of the *t*-score at significance level α of 0.05 (*t*_0.05, 1274_) is 1.962. The absolute value of the test statistic for our example is less than the critical value [(*t*_0.05, 1274_) > −0.061], so we accept the hypothesis as proven. The use of this statistical analysis in the diagnosis, treatment and analysis of XAFS is novel.

Photoreduction of the sample solution through the sample cell was initially observed during the experiment at room temperature. We performed the two-sample *t*-test to statistically measure the change of room-temperature sample measurements with radiation damage. We assume that the measurements are distributed normally. We tested the hypothesis that the measurements are similar during the experiment performed at room temperature before minimizing radiation damage. The *t*-score is 9.758. If equal variances are assumed, then the number of degrees of freedom is 1160. The critical value of the *t*-score at significance level α of 0.05 (*t*_0.05, 1160_) is 1.962. The absolute value of the test statistic for our example is greater than the critical value [(*t*_0.05, 1160_) < 9.758], so we must reject the hypothesis and conclude that the two scans produce inconsistent measurements before the minimization of radiation damage at the 0.05 significance level under electrochemical control at room temperature. Thus, a proper minimization of radiation damage is needed.

In this experiment, the horizontal shutter was narrowed to 0.6 mm so that the intensity at the upstream ion chamber was decreased from 150 000 counts per second to 85 000 counts per second. The fluorescence detector was also moved closer to improve the counting statistics. The low-flux beam was maintained for the dilute Cu^II^:Aβ samples during this experiment. The effect of photoreduction was also minimized by increasing the flow rate of the sample solution while maintaining the data collection. The XAS-EC cell efficiently facilitated the necessary conditions to increase the flow of sample solutions. In these Cu^II^:Aβ experiments, filter banks were used to attenuate the beam to reduce the photodamage.

When the experimental geometry and the sample conditions had been adjusted, strong evidence for non-existence of photodamage was observed during redox experiments at room temperature. Fig. 6 ▸(*b*) shows the XANES spectra of Aβ_4–8/12/16_ peptides at room temperature providing the Cu^II^ features at room temperature before applying redox conditions. We performed the two-sample *t*-test to provide a statistical measure of the consistency of room-temperature sample measurements with minimization of radiation damage. We assumed that the measurements are distributed normally. We tested the hypothesis that the measurements are similar due to the minimization of radiation damage. The *t*-score was −0.072. If equal variances are assumed, then the number of degrees of freedom is 1160. The critical value of the *t*-score at significance level α of 0.05 (*t*_0.05, 1160_) is 1.962. The absolute value of the test statistic for our example is less than the critical value [(*t*_0.05, 1160_) > −0.072]. Therefore we must accept the hypothesis and conclude that the two scans produce consistent measurements after the minimization of radiation damage at the 0.05 significance level under electrochemical control at room temperature.

Summers *et al.* (2019 ▸) raise a very important fourth concern about radiation (beam) damage and photoreduction, especially when past studies are considered. If photoreduction occurs, as discussed briefly above, a lower-coordination site may be found; the signal will be blurred and become more pleomorphic; and parameters such as 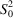 may become anomalously low. Further, the binding may then relate to Cu^I^ instead of Cu^II^. Summers *et al.* (2019 ▸) analysed a series of major past studies (Stellato *et al.*, 2006 ▸; Morante, 2008 ▸; Minicozzi *et al.*, 2008 ▸; Shearer & Szalai, 2008 ▸; Shearer *et al.*, 2010 ▸) and concluded that these were all significantly photoreduced. They also collected repeated (5) scan measurements in their studies for each moiety to a range of *k* ∼ 12 Å^−1^ and initially showed that significant photodamage occurred from the first to the second scan. Later they offset the position 0.5 mm between scans. However, if photodamage occurred within the first scan, as they observed, then the damage would have affected the higher-*k* structure. More effort is sometimes needed to ensure and quantify that radiation damage has not occurred within the first two scans. Flow cells are used for room-temperature studies, but may be used for both room- and low-temperature studies. It is important to quantify any damage within a single scan, otherwise the pre-edge or first few XANES peaks might be the only undamaged data. The consistency of our refined parameters in repeated scans and in combined scans – both merged by weighted means from the uncertainty, and separately by simultaneous fitting of multiple and sequential data sets – to within the uncertainty confirms the absence of radiation damage in the final spectra. This analysis and statistical testing approach appears novel in the literature but is extremely valuable especially for diagnosis and analysis of beam damage, pleomorphism and other sample variability.

## 6. Monochromator glitches in fluorescence

X-rays are simultaneously diffracted from multiple sets of Bragg planes within the monochromator crystal, leading to a sudden decrease or increase in the diffracted intensity towards the experimental setup at discrete energies (Chantler, 1995 ▸; Quintana & Hart, 1995 ▸; Sutter *et al.*, 2016 ▸; Bridges *et al.*, 1991 ▸; Li *et al.*, 1994 ▸; Ascone *et al.*, 2003 ▸). Various methods have been developed to understand the appearance of such glitches and improve their normalization and reduction (Bridges *et al.*, 1991 ▸, 1992 ▸; Li *et al.*, 1994 ▸; Van Der Laan & Thole, 1988 ▸; Tang *et al.*, 2015 ▸). The glitch spectra are mapped out in some XAS beamlines for better experimental measurement. Intensity fluctuations due to X-ray optics should cancel out when calculating the corrected ratio of incident and transmission intensity. If the intensity responses are not linear, then the fluctuations will not be eliminated.

Normalization of the signal by the incident intensities for fluorescence measurements is insufficient to compensate for the glitches (Sutter *et al.*, 2016 ▸). It is challenging to attempt to map glitches for fluorescence measurements in comparison with transmission measurements. Different techniques are used to map glitch energies in beamlines to obtain high-quality measurements. Deleting the corresponding channel, preferably after confirming the origin of the apparent glitch in the fluorescence spectra, is the most common practice in fluorescence XAFS (Ravel, 2016*a* ▸). Recently, Wallace *et al.* (2020 ▸) introduced a algorithm for removing glitches (deglitching)which has several strategies and parameters for transmission and fluorescence mode.

**Figure 7:**
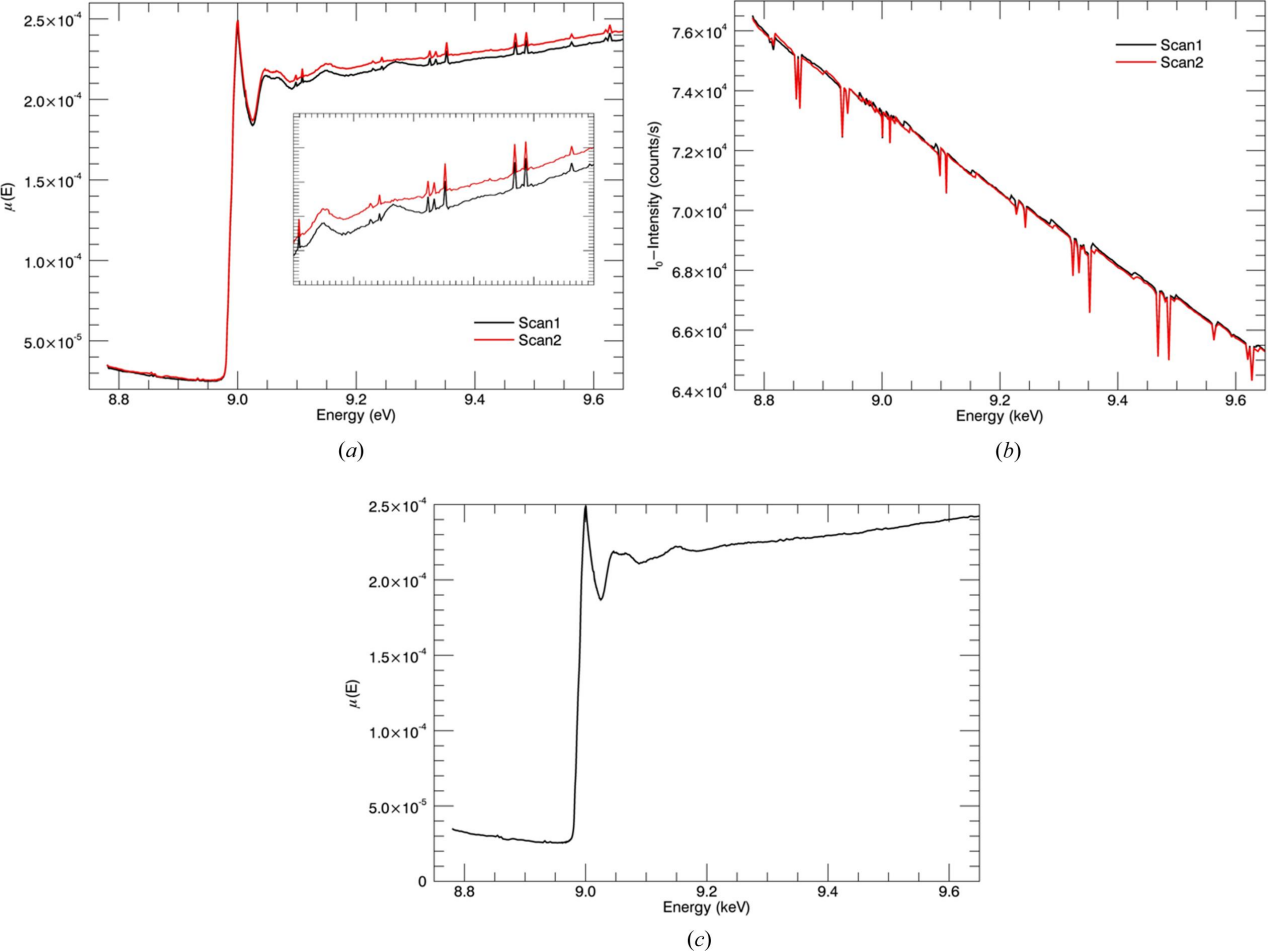
Sudden changes appearing in the intensity versus energy plots (sharp dips) in (*a*) the fluorescence spectra and (*b*) incident intensities *I*_0_ are monochromator glitches or from mirror tuning or detuning. About 90% of the glitches disappear after normalization. Remaining glitches can be due to uncompensated beam crashes, beam filling, motor slips and a change of *I*_0_ loading. Partially corrected or uncorrected glitches after normalization were removed by deleting the corresponding channels. (*c*) The fluorescence spectra after removing monochromator glitches.

Fig. 7 ▸ presents the fluorescence measurements with monochromator glitches. The incident intensity has sharp dips due to multiple diffraction coming from Bragg planes of the monochromator. Two approaches to deglitching are offered by the *ATHENA* software (Ravel & Newville, 2005 ▸). A similar concept was introduced in our preliminary data analysis code for deglitching. In this work, most of them were compensated by normalizing measurements from the incident intensity. Remaining spurious points were then removed [Fig. 7 ▸(*c*)]. Our approach is effective and sufficient but certainly not unique in the literature and also not the best practice. The best practice is perhaps afforded by the X-ray extended range technique (XERT) experiments in transmission where such glitches are normalized and hence have no effect on the data. Unfortunately this is not yet possible in fluorescence measurement.

## 7. Sample heterogeneity with time

The quality of XAS data is reduced due to electronic noise, instability of the X-ray beam, stochastic noise and other artefacts including fluctuations in monochromators and inhomogeneity of the sample (Abe *et al.*, 2018 ▸; Heald, 2015 ▸). The background contribution can be reduced with a decrease in flux, but the features of the actual signal will also be diminished. Another key approach to avoid the involvement of background effects is incorporation of a proper data normalization. Chantler *et al.* (2015 ▸) introduced a hybrid technique to improve statistical counts, resulting in the preservation of significant information in fluorescence XAFS. Sample homogeneity strongly affects the quality of XAFS amplitudes (Goulon *et al.*, 1982 ▸).

### 7.1. Contaminants and background scatter

**Figure 8:**
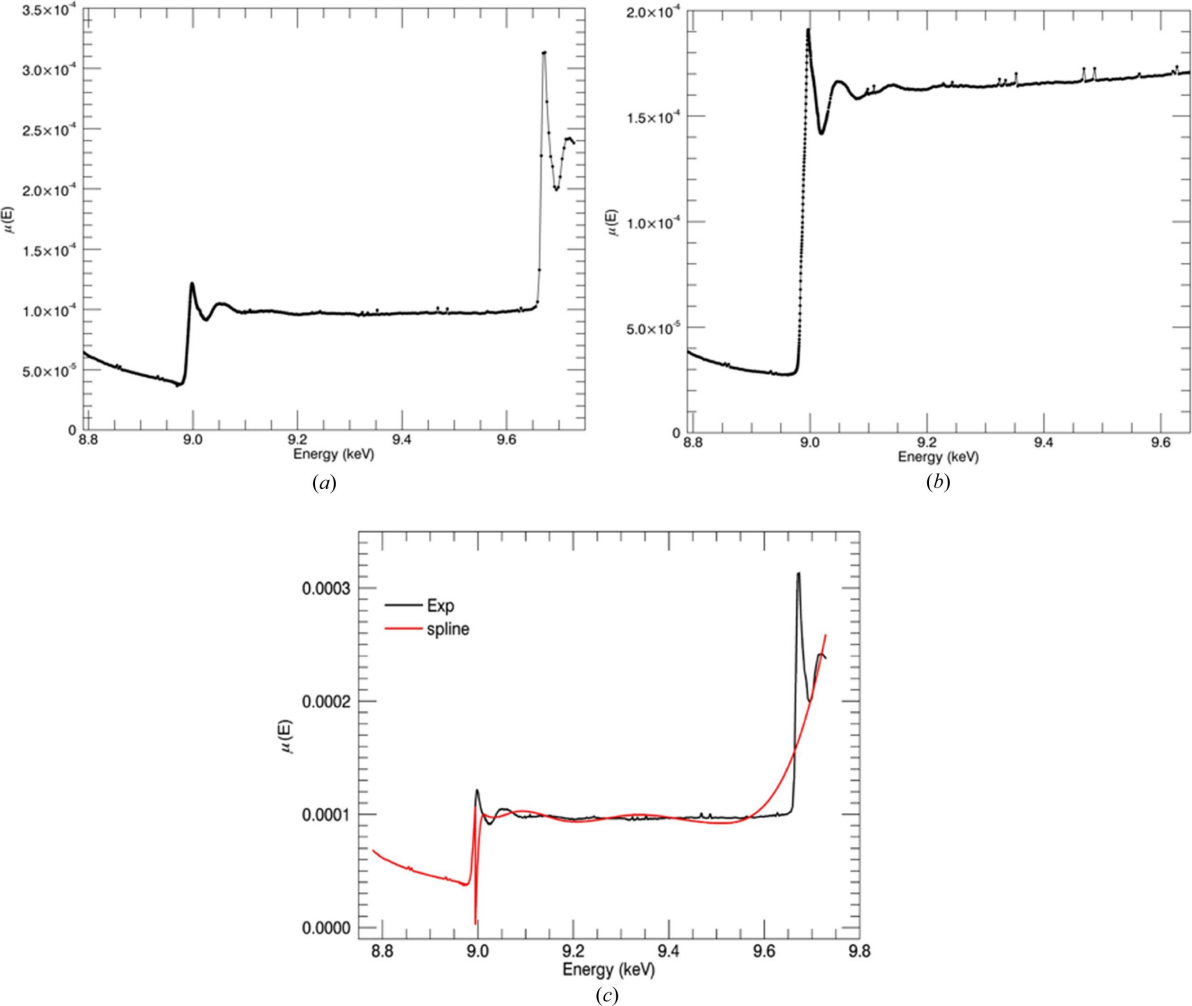
The fluorescence spectrum of the sample with a copper *K* edge. (*a*) The spectrum has an unwanted zinc *K* edge at an energy of 9.66 keV from a background material. (*b*) The spectrum after eliminating the unwanted peak. (*c*) The spline (red) was disrupted by the unwanted edge and it will distort the χ(*k*) spectrum.

The oscillatory part from the fluorescence XAS measurements was extracted to investigate the structure of the sample. Measurements were obtained up to a maximum *k* of 14 Å^−1^ at the beginning of the experiment. However, an unwanted peak appeared at the zinc *K*-absorption edge energy of ∼9.66 keV in the fluorescence spectrum, affecting the spline subtraction and extraction of oscillatory parts. The signal is deteriorated by the background zinc signal. Fig. 8 ▸(*a*) illustrates the spectrum with the unwanted edge at the end of the fluorescence spectrum. Background scattering of fluorescence radiation due to zinc material was observed and characterized in other experiments at the Australian Synchrotron (Ekanayake *et al.*, 2021 ▸; Sier *et al.*, 2022 ▸). In this experiment, the *k* range was simply truncated to eliminate the unwanted peak at the end of the fluorescence spectrum. Then, the main signal was dominant and therefore all the XANES and EXAFS features were significant in the spectrum. Fig. 8 ▸(*b*) shows the measurements after truncating the *k* range for high-quality data.

Fig. 8 ▸(*c*) shows the spline error if the second edge is not trimmed. This would disrupt the data normalization and the χ(*k*) fitting. Implausible structural information would follow from XAFS refinements with a poor spline calculation. The appearance of the unwanted peak at the far end of the spectrum is due to some background scattering coming from a material containing zinc (*Z* = 30). The fluorescence radiation might come from the upstream geometry of the experiment such as ion chamber windows or slits. Another possible explanation for this might be the radiation scattering coming from the filter banks that were used to attenuate the beam flux.

### 7.2. Bubbles

**Figure 9:**
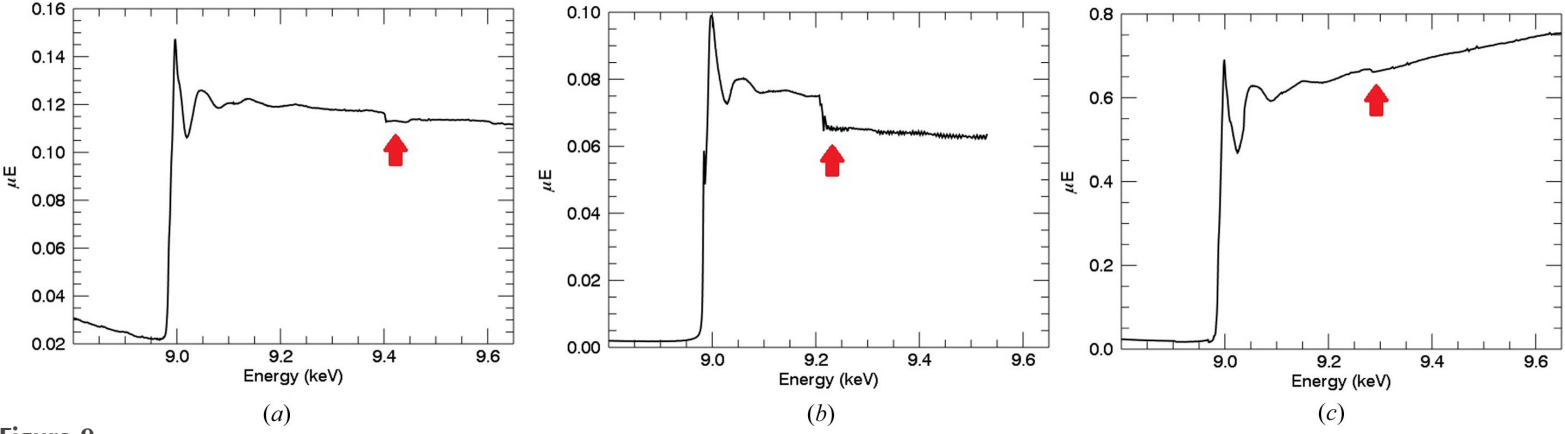
Bubbles were created in the cell due to loose fittings, resulting in a discontinuity over the fluorescence spectrum. Red arrows represent jumps in the spectra. These bubbles were removed by adjusting the solution flow.

The room-temperature XAS-EC cell was maintained under near-physiological conditions. Air bubbles were created in the electrochemical cell setup partially due to loose fittings. The air bubble passed through the cell resulting in a jump in the fluorescence spectrum, as shown in Fig. 9 ▸. The bubbles which were created in the sample solution were removed by adjusting the flow of the solution, and then stable measurements were obtained under pulsed flow conditions. It is important to ensure all tube fittings are sufficiently tight to avoid air bubbles through the sample solution.

### 7.3. Noise at high *k*

The Cu:amyloid-β samples used in this experiment were maintained under near-physiological conditions during the experiment. Therefore, the signal-to-noise ratio was low at higher energies, resulting in frequent fluctuations in the fluorescence spectra and χ(*k*) spectra. Usually, fluorescence XAS measurements are limited to *k* = 12 Å^−1^ or less for dilute samples due to noise at high *k* (Penner-Hahn, 1999 ▸). Fluctuations due to stochastic noise (Hu & Booth, 2009 ▸) and electric noise (Abe *et al.*, 2018 ▸) in the χ(*k*) spectrum at high *k* will distort determined bond lengths and parameters. The unwanted noisy measurements were trimmed to achieve accurate spectral data. Data truncation was carried out before calculating χ(*k*) values as the spline calculations could be disrupted by ambiguous measurements produced at higher energies. Data truncation is not the only solution for dealing with this issue. Careful selection of the spline, pre-edge and normalization ranges is one way to reduce spurious artefacts in the measurements.

## 8. Normalization of fluorescence data

There is a significant dispersion across the spectra in a multi-element fluorescence detector since each pixel generates its own spectrum. The precision of the refined structural parameters is limited by spectral shape distortion caused by the dispersion. The attenuation must decrease with the increase of energy as predicted by theory, whereas the slope after the edge of the fluorescence spectra in the current work and many experimental fluorescence spectra increases with the energy. The fluorescence spectrum produced from each pixel has a variation due to systematic effects of absorption, self-absorption and uncalibrated detector efficiencies in fluorescence (Trevorah *et al.*, 2019 ▸). Previous investigations commented upon distortions of fluorescence X-ray absorption spectra (Goulon *et al.*, 1982 ▸; Troger *et al.*, 1992 ▸; Pfalzer *et al.*, 1999 ▸). A general solution using a software package suitable for a wide range of experiments was introduced by Chantler *et al.* (2012*b* ▸), depending upon the experimental geometry and detector details.

The dispersion issue was resolved by normalizing the signal in a self-consistent method for the measurements obtained in the current work. The pixel scan normalization in the current work was checked using several approaches. All pixel scans were normalized to the average fluorescence measurement of all pixels at three different energy channels. The last energy channel and energies just before and after the edge energy were used as the pinning point for normalization. The normalization at the last energy channel reduces the variance while other options increase the variance mainly above the edge. The normalization factor used to fix the spectral deviations is

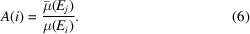

The scaling factor is energy dependent. Here, 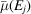 is the average mass attenuation coefficient of all pixel data at pinning energy. μ(*E*_*i*_) is the mass attenuation coefficient at measured energies. *j* and *i* represent the energy position. Then the corrected mass attenuation coefficient is

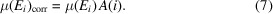

**Figure 10:**
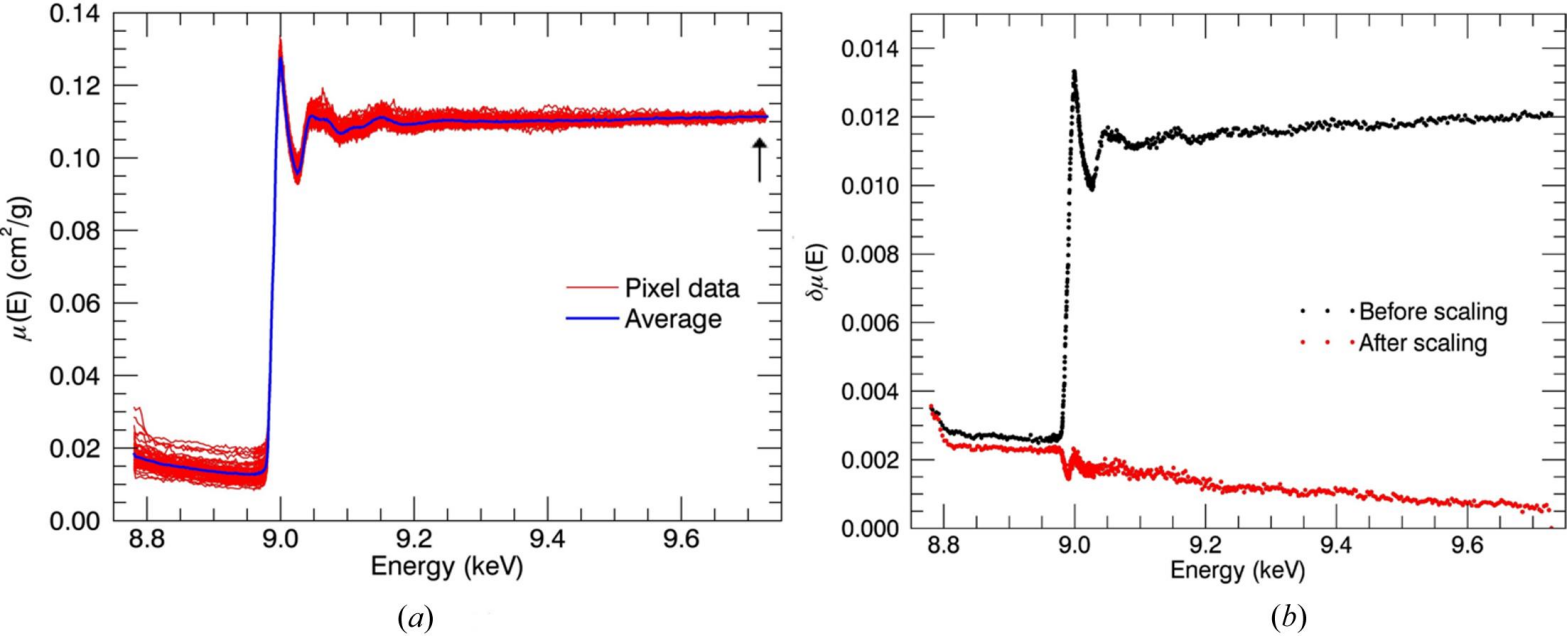
(*a*) Use of the last data point for the normalization decreased the uncertainty by 8% and hence was used to fix the dispersion due to self-absorption and detector inefficiencies. (*b*) The uncertainty of the fluorescence measurements at each energy for the normalization with the last energy point.

Fig. 10 ▸ illustrates the normalization spectra obtained from pixels in the multi-channel detector. The uncertainties of the measurements were increased by about 6% with the normalization when using the point just before the absorption edge, whereas they were decreased by about 8% when using the data points at the end of the spectrum and just after the absorption edge. Using the end point is much more stable than the point just after the absorption edge. The value just after the absorption edge is very sensitive to beam stability and could vary in repeated scans. Therefore, the mass attenuation coefficient at the end of the spectrum was used in normalization.

## 9. Flattening XAFS

Fluorescence XAS shows a characteristic upward-sloping spectrum as the upstream detector becomes less absorbing with the increase of the X-ray beam energy, whereas the fluorescence detector has similar absorption with energy. This detector effect is eliminated by normalization.

**Figure 11:**
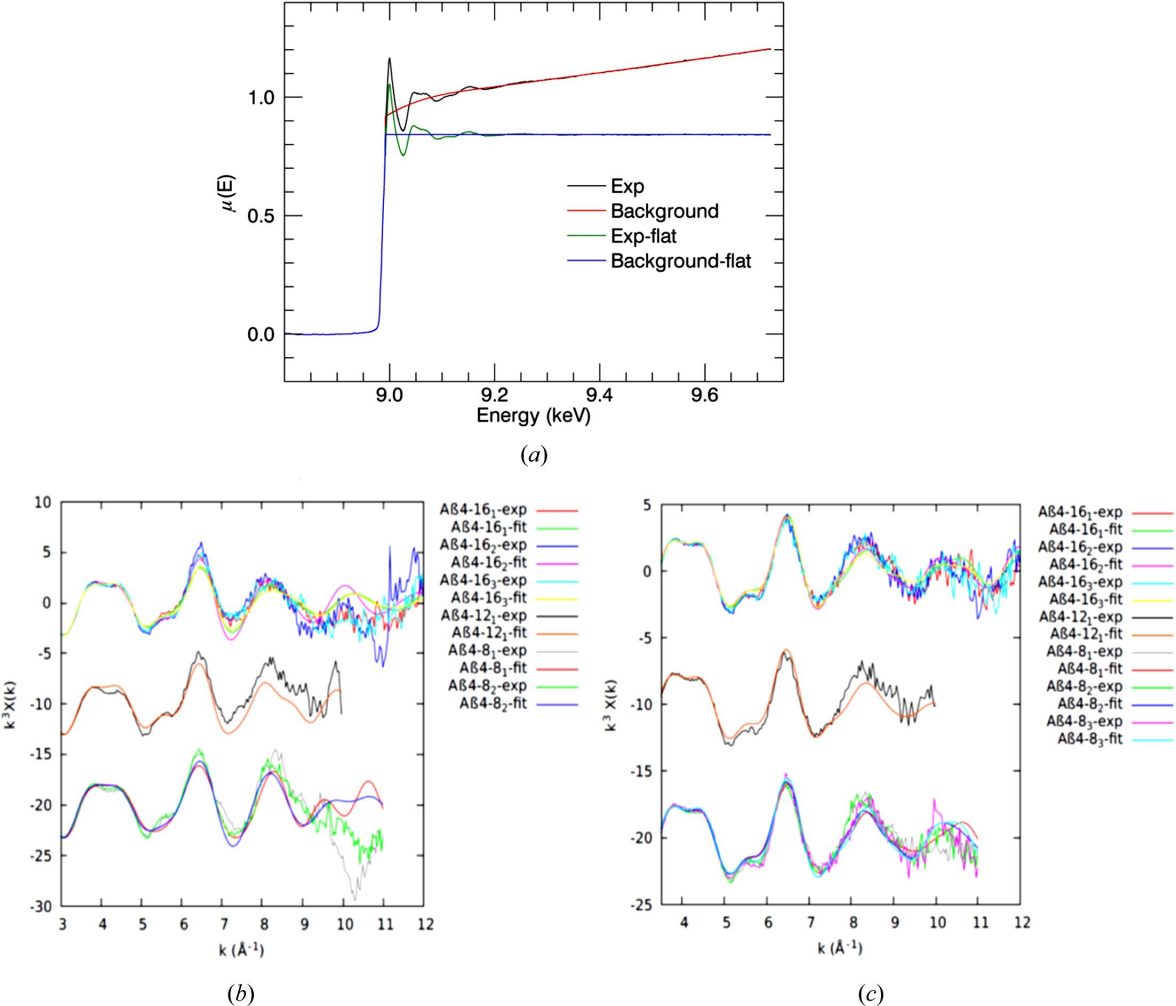
(*a*) Flattened and non-flattened fluorescence measurement interpretation in *Mu2Chi* (Schalken & Chantler, 2018 ▸) for Cu^II^:Aβ_4–16_1__. *k* ^3^χ(*k*) versus *k* for Cu^II^:Aβ_4–16_1,2,3__, Cu^II^:Aβ_4–12_2__ and Cu^II^:Aβ_4–8_1,2,3__ and their fits obtained from *eFEFFit* (*c*) without and (*d*) with normalized measurements. The refined structural parameters of the normalized spectra were reliable and realistic.

If the post-edge spectrum oscillations have an upward trend, then the normalizing procedure is really important. Flattening is a correction of measured data such that the oscillatory part of the measurements is pushed up to unity (Ravel, 2016*b* ▸). It has been suggested that this flattening process does not affect the EXAFS analysis (Kelly *et al.*, 2008 ▸). Our measurements have an upward trend after the edge energy. Fig. 11 ▸(*a*) shows XAS measurements generated from *eFEFFit* for Cu^II^Aβ_4–16_3__. Consistent χ(*k*) oscillations for repeated measurements were obtained from flattened measurements using the *eFEFFit* package. Figs. 11 ▸(*b*) and 11 ▸(*c*) show the *k* ^3^χ(*k*) plots obtained from *eFEFFit* without and with flattened measurements. In *ATHENA* (Ravel & Newville, 2005 ▸), flattening is a default setting that can be manually disabled. Flattened measurements were obtained by fitting a quadratic curve to the normalized measurements after the edge and then subtracting that curve from measurements. The flattening can remove several differences in interpretation of the spectrum. It can eliminate frequency-dependent artefacts over *k* [Figs. 11 ▸(*b*) and 11 ▸(*c*)]. The remaining fluctuations are due to self-absorption and noise. The background plot is an empirical polynomial spline function fitted to the normalized spectra to estimate the isolated atom background curve. Subtraction of the background curve removes background effects from a matrix and solvent and any absorption or scattering from atoms not involved in the edge. Correction for self-absorption, linear combination fits and peak fittings are more promising after the flattening process. χ^2^ was improved by about 50% in XAS fitting using *eFEFFit* with the normalization (or flattening to 1) measurements. This recent evidence suggests that the normalizing has a significant impact on *eFEFFit*-refined outcomes, especially for upward-increasing fluorescence XAS measurements after the edge energy.

## 10. Conclusion

Careful investigation of experimental systematic errors and monitoring of radiation damage are extremely important when collecting fluorescence measurements of biological samples. Correction of the systematics, avoiding photodamage and propagation of experimental uncertainties will provide reliable measurements for precise structural investigations. This permits hypothesis testing.

The XAS-EC setup is appropriate for collecting high-quality XAS measurements of biological samples under near-physiological conditions. The fluorescence spectra of the current experiment were produced from a multi-channel detector. Defective pixel data based on baseline artefacts and sensitivity were manually removed and hence a 15% improvement in structural refinement was achieved. In certain beamlines, the correction of the defective and dead pixels of the fluorescence detector could be performed using control codes during data collection. If the exclusion of defective and dead pixels is impractical during the data collection, introduction of a preliminary analysis code for corrections is required, often currently with manual oversight, as herein.

The effect of the dead time on fluorescence measurement was explored and about a 1–2% change was observed. The beamline controls often include the dead-time corrections, but it is important to examine the correction method. The dead time must be corrected with a reliable formula when analysing data using raw measurements. Almost uniquely, we have investigated both the standard dead time and a second time constant which indeed improved the data analysis further, thanks to the Australian Synchrotron beamline team and the *Sakura* data pre-processing code.

Monochromator glitches were identified and removed for all samples. Normalization of data by incident intensity removed 90% of the glitches. Omitted glitches must be removed by deleting the corresponding channels. This is unfortunately manual in most experiments, as herein, though some automated approaches exist, as for example in *ATHENA*; however, we recommend manual oversight or the much more advanced predictive algorithm used at Diamond.

Unwanted noisy measurements at higher *k* were truncated and therefore XAS measurements were limited to *k* = 10 Å^−1^. Selection of spline, pre-edge subtraction and post-edge normalization is also important to achieve better spectra at higher *k*. Refinement of structural parameters from EXAFS is compromised with a poor selection of an incorrect spline. Therefore, selection of the measurement range prior to the data collection and extraction of XAFS oscillations must be carefully investigated.

Dispersion of spectra due to sensitivity differences in individual channels was handled by normalizing signal in a self-consistent method in the current work. The uncertainty of the measurements decreased by about 8% with the normalization of the data using the end point of the spectrum. The selection of a scale for normalization is critical, and we proved for this experiment that the normalization is more stable when using the end point of the spectrum, rather than when using a point following the absorption edge, or of course by not normalizing at all. However, the dispersion of spectra due to self-absorption is not completely corrected by implementing this method, and it could be performed with a much more detailed experimental data collection approach, such as that used by Trevorah *et al.* (2019 ▸).

Further corrections, such as dark current subtraction and blank normalization, should be carried out for the fluorescence measurements because electronic noise and background signals can always have a considerable impact on the measurements. Another systematic correction that we ought to perform is energy bandwidth correction. A monochromator could produce an error in energy selection based on a range of factors including the distribution of the lattice spacing of the monochromator under thermal stress, the acceptance angle and the divergence of the incident beam. This would introduce a change in the collected measurements. The remaining corrections can be investigated and corrected with careful collection of additional measurements.

Assumptions made in fitting the EXAFS data were minimized by investigating and quantifying as many systematic errors as possible. Interpolations of experimental data on to a regularly spaced grid in *k* space will distort experimental values, information content, point density and experimental uncertainties (Schalken & Chantler, 2018 ▸). In this study, oscillation extractions were performed by avoiding interpolations in *k* space to get more insightful results for hypothesis testing. Fitting was done in *k* space as an interpolation to a uniform grid is required to obtain a Fourier transform for fitting in *r* space. The fit was performed without any *k* weighting to avoid emphasizing different regions of the spectrum.

Radiation damage of the sample was explicitly diagnosed and minimized during the data collection. This is strongly supported by the consistency of the repeated measurements and the improved structural parameters. The two-sample *t*-test was used to statistically measure the consistency of the measurements during data collection. This could be easily performed for any number of repeated scans of a sample. If the photodamage is not properly identified, fitting EXAXFS data will be complicated and, therefore, the fitted parameter will provide misleading structural information. We recommend this significant advance in all radiation-fragile systems, and also anywhere there is potential for pleomorphism and other heterogeneity.

The estimation of all the specified systematic corrections can be carried out for a single scan; therefore, they can all be automated for any number of repeated fluorescence scans of the material. In this work, the propagation of uncertainties was obtained from the pointwise variance of the spectra. Standard errors of the fluorescence measurement at each energy point produced an uncertainty. A reliable goodness-of-fit value can be evaluated by utilizing experimentally propagated uncertainties and hence a robust investigation of structural information of Cu-bound amyloid-β peptide samples can be carried out using *eFEFFIT* (Ekanayake *et al.*, 2023 ▸).
